# Supplementary material for: Effects of Exercise on Patients with Obstructive Sleep Apnea: A Systematic Review and Meta-Analysis
Source: Int J Environ Res Public Health. 2022 Aug 31;19(17):10845. doi: 10.3390/ijerph191710845 (PMC9518429; doi:10.3390/ijerph191710845)
Supplement: Supplementary file 1 [file ijerph-19-10845-s001.zip › ijerph-1851623-supplementary.pdf]

Supplementary Materials

PubMed search strategy

#1 apneas, obstructive sleep
#2 obstructive sleep apneas
#3 sleep apneas, obstructive [Mesh]
#4 obstructive sleep apnea syndrome
#5 obstructive sleep apnea
#6 OSAS
#7 Syndrome, Sleep Apnea, Obstructive
#8 Sleep Apnea Syndrome, Obstructive
#9 Apnea, Obstructive Sleep
#10 Sleep Apnea Hypopnea Syndrome
#11 Syndrome, Obstructive Sleep Apnea
#12 Upper Airway Resistance Sleep Apnea Syndrome
#13 Syndrome, Upper Airway Resistance, Sleep Apnea
#14 #1 OR #2 OR #3 OR #4 OR #5 OR #6 OR #7 OR #8 OR #9 OR #10 OR #11 OR #12 OR #13
#15 Exercise [Mesh]
#16 Physical Activity
#17 Activities, Physical
#18 Physical Activities
#19 Exercise, Physical
#20 Acute Exercise
#21 Exercise, Acute
#22 Isometric Exercise
#23 Exercise, Isometric
#24 Exercise, Aerobic
#25 Aerobic Exercise
#26 Exercise Training
#27 #15 OR #16 OR #17 OR #18 OR #19 OR #20 OR #21 OR #22 OR #23 OR #24 OR #25 OR #26
#28 randomized controlled trial
#29 randomized
#30 placebo
#31 #28 OR #29 OR #30
#32 #14 AND #27 AND #31

EBSCO search strategy

#S1 AB Obstructive sleep apnea OR AB Apneas, Obstructive Sleep OR AB Obstructive Sleep Apneas OR AB Sleep Apneas, Obstructive OR AB Obstructive Sleep Apnea Syndrome OR AB Obstructive Sleep Apnea OR AB OSAHS OR AB Syndrome, Sleep Apnea, Obstructive OR AB Sleep Apnea Syndrome, Obstructive OR AB Apnea, Obstructive Sleep OR AB Sleep Apnea Hypopnea Syndrome OR AB Syndrome, Obstructive Sleep Apnea
#S2 AB Syndrome, Upper Airway Resistance, Sleep Apnea OR AB Upper Airway Resistance Sleep Apnea Syndrome
#S3 AB Exercise OR AB Exercises AND AB Physical Activity AND AB Activities, Physical AND AB Activity, Physical AND Physical Activities AND Exercise, Physical AND Exercises, Physical AND Physical Exercise AND Physical Exercises AND Acute Exercise AND Acute Exercises
#S4 AB Exercise, Acute OR AB Exercises, Acute AND AB Exercise, Isometric AND AB Exercises, Isometric AND AB Isometric Exercises AND Isometric Exercise AND Exercise, Aerobic AND Aerobic Exercise AND Aerobic Exercises AND Exercises, Aerobic AND Exercise Training AND Exercise Trainings
#S5 AB Training, Exercise OR AB Trainings, Exercise
#S6 S1 OR S2
#S7 S3 OR S4 OR S5
#S8 S6 AND S7
#S9 AB randomized controlled trial OR TI randomized controlled trial OR TX randomized controlled trial OR AB randomized OR TI randomized OR TX randomized OR AB placebo OR TI placebo OR TX placebo
#S10 S8 AND S9

Web of Science search strategy

#1 TS=(Apneas, Obstructive Sleep OR Obstructive Sleep Apneas OR Sleep Apneas, Obstructive OR Obstructive Sleep Apnea Syndrome OR Obstructive Sleep Apnea OR OSAHS OR Syndrome, Sleep Apnea, Obstructive OR Sleep Apnea Syndrome, Obstructive OR Apnea, Obstructive Sleep OR Sleep Apnea Hypopnea Syndrome OR Syndrome, Obstructive Sleep Apnea OR Upper Airway Resistance Sleep Apnea Syndrome OR Syndrome, Upper Airway Resistance, Sleep Apnea OR Sleep Apnea, Obstructive)
#2 TS=(Exercises OR Physical Activity OR Activities, Physical OR Activity, Physical OR Physical Activities OR Exercise, Physical OR Exercises, Physical OR Physical Exercise OR Physical Exercises OR Acute Exercise OR Acute Exercises OR Exercise, Acute OR Exercises, Acute OR Exercise, Isometric OR Exercises, Isometric OR Isometric Exercises OR Isometric Exercise OR Exercise, Aerobic OR Aerobic Exercise OR Aerobic Exercises OR Exercises, Aerobic OR Exercise Training OR Exercise Trainings OR Training, Exercise OR Trainings, Exercise)
#3 TS=(randomized controlled trial OR randomized OR placebo)
#4 #1 and #2 and #3

CNKI search strategy

#1 主题=（阻塞性睡眠呼吸暂停 or 阻塞性睡眠呼吸暂停低通气综合征 or 阻塞性睡眠呼吸暂停 or 阻塞性呼吸暂停 or 阻塞性呼吸睡眠暂停综合征 or 阻塞型睡眠呼吸暂停）
#2 主题=（有氧运动 or 训练 or 抗阻 or 运动 or 有氧运动 or 有氧代谢 or 耐力性运动 or 耐力运动）

#3 文献类型=（研究论文）
#4 #1 and #2 and #3

CNKI search strategy (in English)

#1 theme = (obstructive sleep apnea or obstructive sleep apnea hyponea syndrome or obstructive sleep apnea syndrome or OSA or OSAS or OSAHS)
#2 theme = (aerobic or training or resistance or exercise or aerobic exercise or endurance exercise)
#3 article type = (Randomized Controlled Trial)
#4 #1 and #2 and #3

VIP search strategy

#1 主题=（阻塞性睡眠呼吸暂停 or 阻塞性睡眠呼吸暂停低通气综合征 or 阻塞性睡眠呼吸暂停 or 阻塞性呼吸暂停 or 阻塞性呼吸睡眠暂停综合征 or 阻塞型睡眠呼吸暂停 or obstructive sleep apnea or obstructive sleep apnea syndrome or patients with obstructive sleep apnea）
#2 主题=（有氧运动 or 训练 or 抗阻 or 运动 or 有氧运动 or 有氧代谢 or 耐力性运动 or 耐力运动 aerobic exercise or aerobic exercises or the aerobic exercise）
#3 #1 and #2

VIP search strategy (in English)

#1 theme = (obstructive sleep apnea or obstructive sleep apnea hyponea syndrome or obstructive sleep apnea syndrome or OSA or OSAS or OSAHS)
#2 theme = (aerobic or training or resistance or exercise or aerobic exercise or endurance exercise)
#3 #1 and #2
